# Comparison of MR Enteroclysis with Colonoscopy in Crohn's Disease-First Locust Bean Gum Study from Turkey

**DOI:** 10.4103/1319-3767.56104

**Published:** 2009-10

**Authors:** Burcu Narin, Faik Sungurlu, Aysun Balci, Alper Arman, Oya O. Kurdas, Masum Simsek

**Affiliations:** Department of Radiology, Haydarpasa Numune Education and Research Hospital, İstanbul, Turkey; 1 Department of Gastroenterology, Haydarpasa Numune Education and Research Hospital, İstanbul, Turkey

**Keywords:** Colonoscopy, Crohn, gadolinium, MR enteroclysis, small bowel

## Abstract

**Background/Aim:**

The aim of this study was to compare magnetic resonance enteroclysis (MRE) findings with those of colonoscopy, using locust bean gum (LBG) as an oral contrast agent in the diagnosis and follow-up of patients with Crohn's disease.

**Materials and Methods:**

Nine patients with histologically proven Crohn's disease were enrolled in this study; MRE was performed within a week of colonoscopy. All patients were examined using a 1.5 T MR Scanner after *per os* administration of 850 mL of a combination of LBG and mannitol. After intravenous administration of 50 mg Eritromisin and 40 mg Scopolamine, images were obtained using a T2-weighted, balanced GRE, fat-suppressed T1-weighted sequence, before and after intravenous gadolinium administration. Bowel wall thickness and enhancement of inflamatory bowel wall were measured.

**Results:**

The oral ingestion of LBG was well tolerated and allowed optimal small and large bowel distention in all patients. MR findings correlated with the colonoscopy results. Additional inflammatory lesions of the colon and mesenteric inflamatory changes such as lymphadenopathy, conglomerate tumor, and fistulas were demonstrated. Contrast enhancement of the affected bowel wall was markedly increased and positive correlation was obtained between bowel wall enhancement and bowel wall thickness.

**Conclusion:**

Gadolinium-enhanced MRE with oral locust bean gum is very efficient in the detection and follow-up of the intestinal and extraintestinal findings of Crohn's disease.

Crohn's disease is a chronic inflammatory disease of the whole gastrointestinal tract which can involve any part from the mouth to the anus. The common sites of inflammation are the terminal ileum and the proximal colon.

Magnetic resonance enteroclysis (MRE) is recognized as a noninvasive imaging method for the detection and identification of small bowel inflammatory disease. MR imaging of patients with Crohn's disease requires an optimal distention of the small bowel, providing the depiction of mural anomalies. Several oral contrast agents have been studied because insufficient contrast between the bowel and the adjacent organ or pathologic lesions decreases image quality. Oral contrast agents can be classified as positive, negative, or biphasic contrast agents, depending on their signal intensity within the lumen of the bowel.

In this study, we used a combination of locust bean and mannitol as an oral contrast agent. The purpose of this study was to evaluate optimal small bowel distention without any ionizing radiation exposure.

## MATERIALS AND METHODS

Nine patients (age: 21-45; average: 30.5 years; gender ratio M:F = 2:7) were enrolled into this study. All patients were examined by using MR enteroclysis within a week of colonoscopy; Crohn's disease was histologically proven in all patients. MR imaging was performed using a 1.5 T system MR Scanner (Philips Intera Achieva; Philips Medical Systems, Holland). All patients drank 850 mL of a combination of 2.5% Mannitol and 0.2% Locust bean gum (LBG) (Roeper, Hamburg) within 45 minutes. This was followed by intravenous administration of 50 mg of Eritromisin (Eritromycin lactobionat; Abbott Pharmaceutics Wiesbaden) after 150 mL of oral contrast solution for the relaxation of pylorus and enhancement of gastric emptying. Prior to imaging, 40 mg Scopolamin was intraveously applied to acquire images free from motion artefacts.

The MR protocol included an axial-balanced GRE sequence (TR/TE/flip angle-3.5/1.7/80 with a slice thickness of 5 mm) in a prone position. Images were also acquired in the sagittal and coronal planes, including the entire abdomen between the diaphragm superiorly and the symphysis pubis inferiorly. Contrast-enhanced imaging was performed after the administration of 0.2 mmol/kg Gd-BOPTA (Multihance, Bracco, Italy). A T1-weighted, 3-D, T1 FFE sequence was perfomed with fat saturation prepulses while the patients held their breath before and after injection of contrast agent as native, arterial (start delay of 30 sec) and venous phases (start delay of 75 sec), with a TR/TE/flip angle-4.7/2.3/10 and a slice thickness of 7 mm.

Segments of the bowel were accepted to be pathological if the wall thickness was > 3 mm and/or showed increased enhancement after the administration of intravenous gadolinium. The maximal signal intensity from the abnormal bowel wall was calculated by using the maximal bowel wall thickness (in millimeters) measured with a distance- measuring tool in a region of interest.

The signal intensity (SI) was measured on coronal T1- weighted images for each abnormal bowel segment in the native, arterial, and venous phases. The SI differences of the pathological bowel wall were compared between the arterial and native phases and venous and native phases using the Pearson's correlation test. Images were analyzed by two independent radiologists (B.N. and F.S.) who were unaware of the colonoscopy results.

## RESULTS

**Figure 1a,b:**
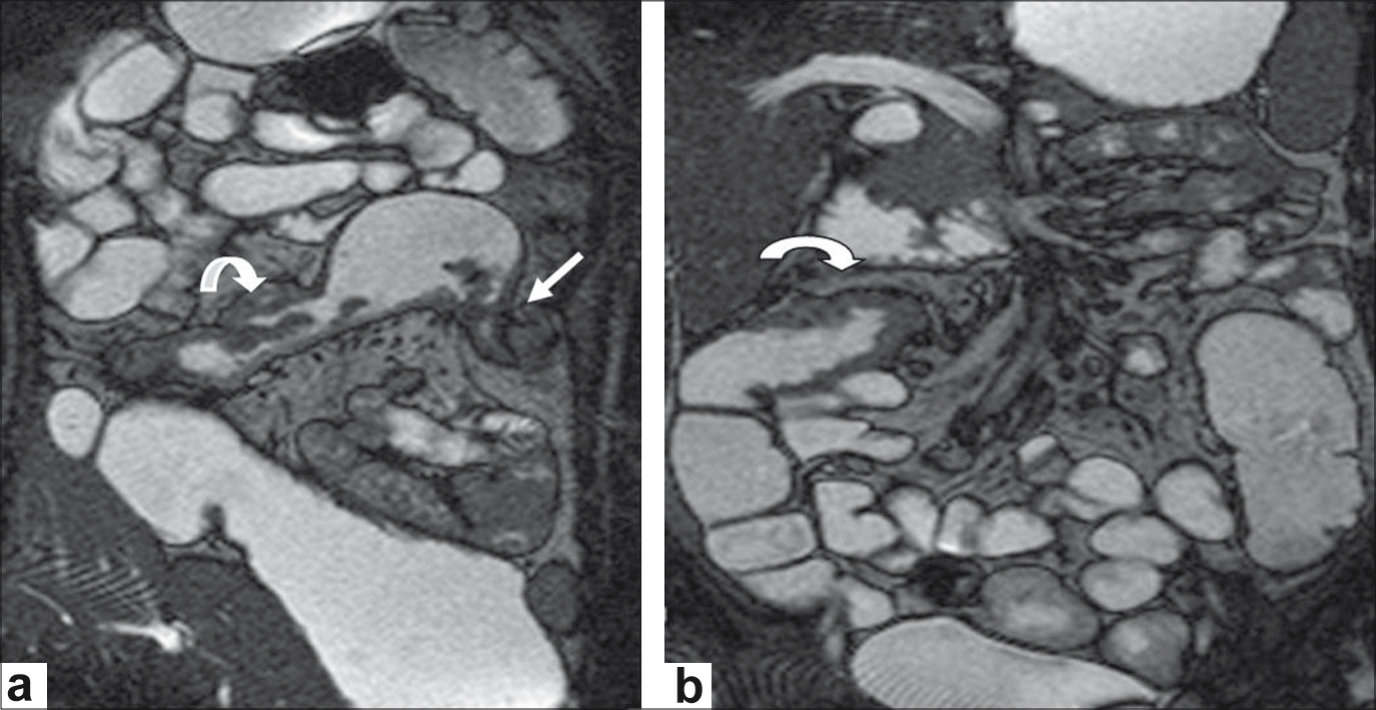
A 26 year-old male patient with Crohn's disease complaining of recurrent abdominal pain. Complete colonoscopy was not possible due to stenosis of the splenic flexura. Coronal balanced GRE(TR/TE/flip angle-3.5/1.7/80) (a) shows severe lumen narrowing of the splenic flexura (straight white arow) with prestenotic dilatation, wall thickening of the transverse colon (curved arrow) (b)

**Figure 2:**
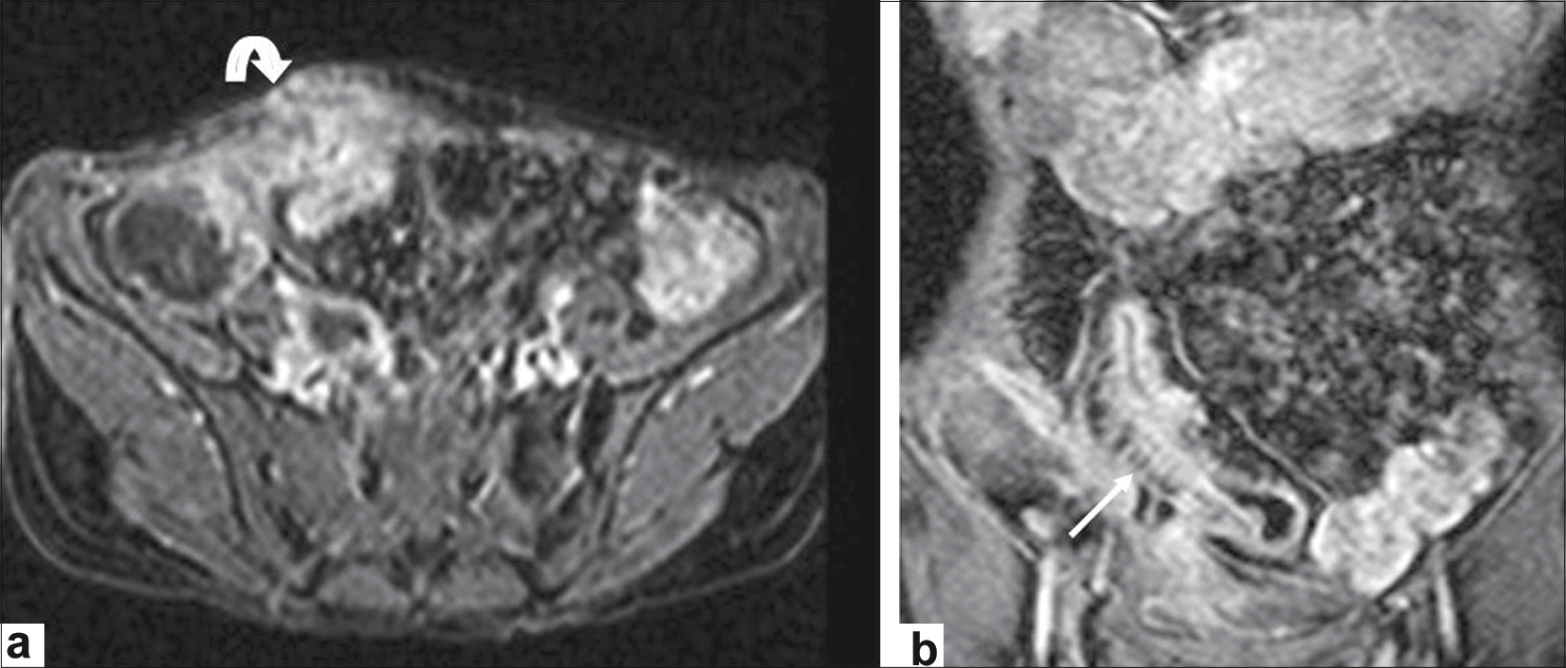
Axial and coronal MR images show (a) diffuse wall thickening and (b) increased gadolinium enhancement of the terminal ileum with fistulas between terminal ileum-bladder (straight arrow) and abdominal wall (curved arrow in a)

MR findings correlated with the colonoscopy results in 8/9 patients. Complete colonoscopy was not possible due to stenosis of the splenic flexura in one patient. MRI showed contrast enhancement with severe lumen narrowing of the splenic flexura, prestenotic dilatation, and inflammation of the distal transverse colon with wall thickening in this patient [Figure 1]. Conglomerate tumor formation of ileal segments and fistulas were detected between terminal ileum and bladder, and terminal ileum and abdominal wall [Figure 2].

**Figure 3:**
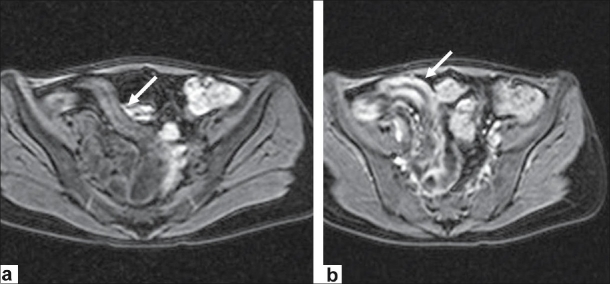
A 32 year-old male patient with Crohn's disease complaining of recurrent episodes of abdominal pain, diarrhea and weight loss. (a) Native and (b) postgadolinium axial FFE T1 images show the diffuse wall thickening and contrast enhancement of the terminal ileum (arrows)

**Figure 4:**
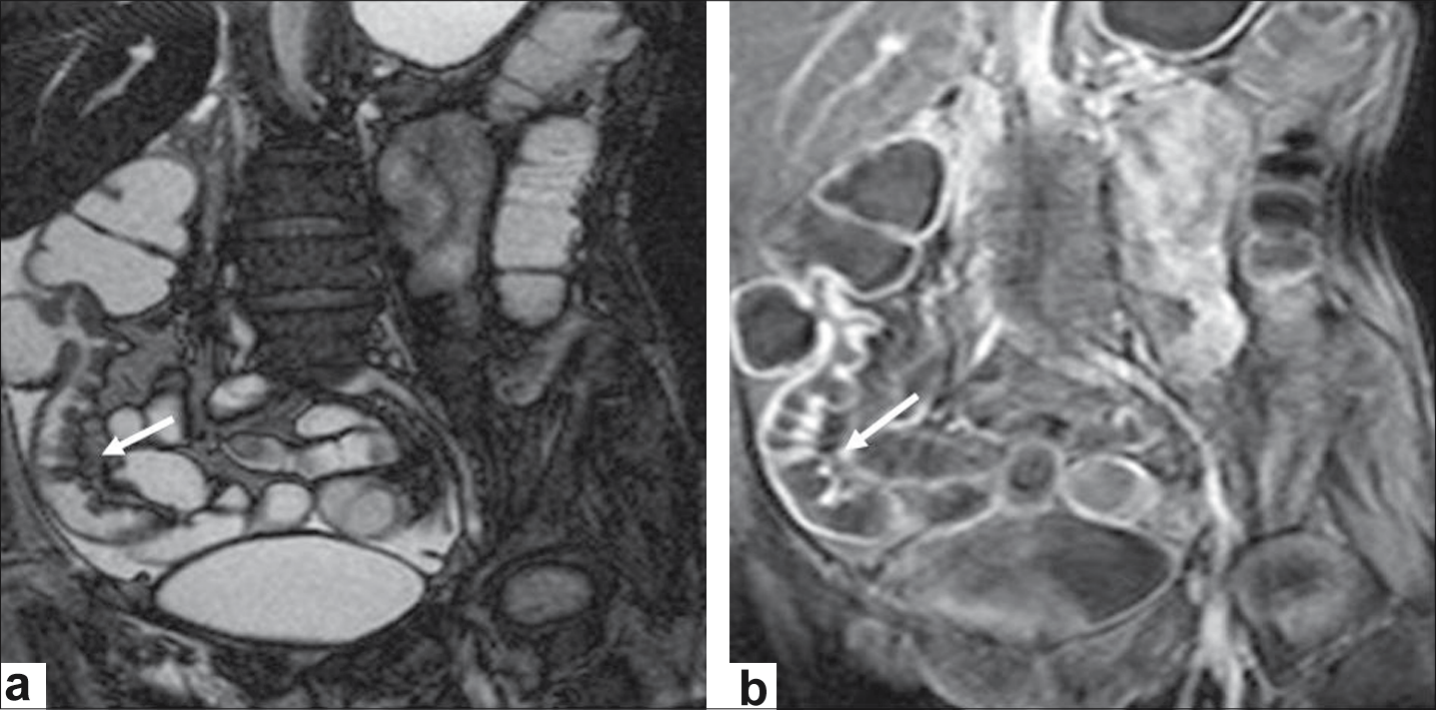
A 40 year-old female patient with persistent diarrhea, crampy abdominal pain, fever, and rectal bleeding. (a) Coronal balanced GRE and (b) coronal FFE T1-weighted image after intravenous gadolinium administration demonstrate the irregularity of the terminal ileum with contrast enhancement (arrows)

All segments with increased contrast enhancement showed high signal intensity values in the arterial phase but a much lower signal increase of SI in the venous phase [Table 1, Figures 3 and 4]. The SI values were 825 ± 180.6 in the native phase, 1602 ± 316.9 in the arterial phase, and 1562 ± 364.5 in the venous phase. All the parameters were compared using Pearson's correlation test; statistical significance was set at *P* < 0.05 with *P* = 0.008 was considered to be statistically highly significant.

**Table 1:** Signal intensity of the bowel wall for precontrast (native) and postcontrast arterial and venous phases, and bowel wall thickness

Patient	Native	Arterial phase	Venous phase	Bowel wall thickness	Arterial-native phase difference	Venous-native phase difference
1	689	1606	1379	10	917	690
2	640	1602	1729	11	962	1089
3	863	1574	1620	8	711	757
4	720	1231	1107	6	501	387
5	840	1519	1135	6.9	679	295
6	923	1660	1562	6	737	639
7	825	1667	1715	14	842	890
8	915	1927	1807	22	1012	892
9	349	815	720	6.5	466	371
Mean	751	1511	1419	10	759	667
Minimum	349	815	720	6	466	295
Maximum	923	1927	1807	22	1012	1089

The signal intensity difference between arterial and native phases, and venous and native phases are calculated by substraction of the respective SI values

## DISCUSSION

MR enteroclysis is a noninvasive imaging method used in the visualization of the entire small bowel with the aid of new contrast agents. Various kinds of intraluminal contrast agents have been used in MR imaging and are classified as positive, negative, or biphasic according to their intraluminal signal intensity.[1,2] Major positive contrast agents are gadolinium chelates, ferrous ammonium citrates, and manganese chloride. Negative contrast agents include oral magnetic particles (OMP), barium sulfate (BaSO_4_), and perflubron. Water, methylcellulose, and polyethylene glycol solution (PEG) are examples of biphasic contrast agents.

An optimal contrast agent should provide homogeneous lumen opacification, high contrast resolution between the lumen and bowel wall, and have low cost and minimal mucosal absorption without causing any adverse effects or artifact formation.[3]

Biphasic contrast agents have been found to be more effective in assessing pathologies of the bowel wall than of fatty tissue.[4] The intestinal lumen is bright against a darkened bowel wall on T2-weighted images whereas the bowel loops appear dark on T1-weighted images. This enables the detection of bowel wall pathologies after the intravenous administration of paramagnetic contrast agents.

Polyethylene glycol solution is a nonabsorbable biphasic contrast agent that provides good distention of the small bowel loops from the jejenum to the terminal ileum. Its disadvantage is its rapid transit time which limits the examination time to under 30 minutes. The increased peristalsis causes motion artefacts and diarrhea.[5,6] Methylcellulose, another biphasic contrast agent, can not be ingested orally and requires positioning of a nasoduodenal tube. Many patients find this method to be uncomfortable and nausea and vomiting have been reported during MR imaging.[6]

As an oral biphasic contrast agent, water is safe and inexpensive but its major limitation is its rapid absorbability in the small bowel; which causes inadequate visualisation of the ileocecal region in more than 30% of the patients.[6] All portions of the small bowel and the ileocecal region can be maximally dilated with the addition of an osmotic substance (mannitol) and a nonosmotic substance (Locust bean gum, LBG).

LBG is extracted from the seeds of the carob tree (*Ceratonia siliqua*). LBG is a galactomannan that is used for its thickening properties in ice creams, dairy gels, and canned products. The addition of LBG, water, and mannitol provides a better distention of the small bowel and the ileocecal region than does a water and mannitol combination and reduces the incidence of diarrhea as a side effect of mannitol.[7] LBG also reduces plasma cholesterol levels.

The combination of water, mannitol, and LBG used in this study resulted in an optimal distention of the small bowel and ileocecal region. In addition to bowel wall pathology; mesenteric inflammatory changes such as fibrofatty proliferation, mesenteric lymphadenopathy, and the existence of conglomerate tumor and fistulas, and sinus tracts were revealed.

As proved in other studies, the signal intensity increase of the bowel wall between the arterial and native phases were found to be significantly correlated with the inflammation and thickening of the bowel wall. The reason for this increased enhancement of the bowel wall was i) the increased uptake of contrast agent by higher mural flow, ii) increased capillary permeability, and iii) increased interstitial deposit.[8–10]

Conventional enteroclysis was found to be the standard imaging method for the detection or follow-up of Crohn's disease. However, it gives only indirect information about the bowel wall and the surrounding structures.[11] The effectiveness of this method is also limited by overlapping of the bowel loops.[12] In addition to conventional enteroclysis imaging, colonoscopy provides the possibility to recognize early mucosal and submucosal changes. Colonoscopy is not a preferred method due to the limitation of the terminal ileum in up to 40% of the patients.

In addition to terminal ileum and ileocecal region imaging, the main advantages of MRI are to demonstrate the more proximal parts of the small bowel, especially the jejenum that can not be reached by colonoscopy. MR enteroclysis also provides other advantages such as the avoidance of ionizing radiation, noninvasiveness, and the ability to show the mesentery and parenchymal organs at the same time. MR enteroclysis should be the first choice among all imaging methods, especially in the follow-up of young patients with Crohn's disease.
